# Micro RNA based MSC EV engineering: Targeting the BMP2 cascade for bone repair

**DOI:** 10.3389/fcell.2023.1127594

**Published:** 2023-02-08

**Authors:** Chun-Chieh Huang, Miya Kang, Kasey Leung, Yu Lu, Sajjad Shirazi, Praveen Gajendrareddy, Sriram Ravindran

**Affiliations:** ^1^ Department of Oral Biology, University of Illinois Chicago, Chicago, Illinois, United States; ^2^ Department of Periodontics, College of Dentistry, University of Illinois Chicago, Chicago, Illinois, United States

**Keywords:** mesenchymal stem cell, extracellular vesicles, bone repair, miRNA, EV engineering

## Abstract

Mesenchymal stem cell derived extracellular vesicles (MSC EVs) possess excellent immunomodulatory and therapeutic properties. While beneficial, from a translational perspective, extracellular vesicles with consistent functionality and target specificity are required to achieve the goals of precision medicine and tissue engineering. Prior research has identified that the miRNA composition of mesenchymal stem cell derived extracellular vesicles contributes significantly towards extracellular vesicles functionality. In this study, we hypothesized that mesenchymal stem cell derived extracellular vesicle functionality can be rendered pathway-specific using a miRNA-based extracellular vesicles engineering approach. To test this hypothesis, we utilized bone repair as a model system and the BMP2 signaling cascade as the targeted pathway. We engineered mesenchymal stem cell extracellular vesicles to possess increased levels of miR-424, a potentiator of the BMP2 signaling cascade. We evaluated the physical and functional characteristics of these extracellular vesicles and their enhanced ability to trigger the osteogenic differentiation of naïve mesenchymal stem cell *in vitro* and facilitate bone repair *in vivo.* Results indicated that the engineered extracellular vesicles retained their extracellular vesicles characteristics and endocytic functionality and demonstrated enhanced osteoinductive function by activating SMAD1/5/8 phosphorylation and mesenchymal stem cell differentiation *in vitro* and enhanced bone repair *in vivo.* Furthermore, the inherent immunomodulatory properties of the mesenchymal stem cell derived extracellular vesicles remained unaltered. These results serve as a proof-of-concept for miRNA-based extracellular vesicles engineering approaches for regenerative medicine applications.

## Introduction

Mesenchymal stem cells (MSCs) possess excellent therapeutic potential. Their immunomodulatory and regenerative potential is well documented across multiple tissues (Kangari et al., 2020), organ systems (Abdi et al., 2008; Yan et al., 2013; Goenka et al., 2020) and diseases (Guo et al., 2020; Hmadcha et al., 2020; Lim and Khoo, 2021; Paris et al., 2021). As a result, MSCs have been explored extensively in clinical trials (Jin and Lee, 2018). However, their translation to mainstream medicine faces several hurdles. For example, as an autologous source, MSCs are limited by quantity, age and disease status of the patient. From an allogenic perspective, screening, testing and banking issues serve as significant limitations.

The beneficial effects of human MSC (HMSC) therapy are attributable to paracrine effects of the MSC secretome (Dai et al., 2007; Gnecchi et al., 2008; Yao et al., 2015). More specifically, MSC derived EVs have been implicated as the drivers behind the effects of the MSC secretome (Lai et al., 2010; Mokarizadeh et al., 2012; Reis et al., 2012). These MSC EVs, as indicated in our recent review, have high therapeutic potential for treatment of dental diseases and for craniofacial tissue regeneration (Cooper et al., 2019). In our published research, we have demonstrated that EVs from lineage-defined MSC induce lineage-specific changes in target naïve MSCs (Bartel, 2004; Narayanan et al., 2016; Huang et al., 2019). This important discovery by our group has since been confirmed by others (Cui et al., 2016; Martins et al., 2016; Zhang et al., 2016). A recent study by Narayanan et al. indicates that MSC EV function supersedes the extracellular matrix (ECM) derived signals, further suggesting the importance of EV signaling and its potential in regenerative medicine applications (Narayanan et al., 2018).

The EV cargo is composed of proteins, mRNAs, miRNAs and other small non-coding RNAs (Thery et al., 2002). The miRNA cargo of EVs is representative of its parental cell status and the differentiated state of the parental MSC confers lineage defining properties to the derivative EVs (Huang et al., 2020a). Dicer and Argonaute2 are two important proteins involved in the generation and functionality of mature miRNAs (Bartel, 2004). Recently, Shirazi et al. demonstrated that in MSCs with Dicer or Argonaute2 suppression, differentiation potential is significantly reduced highlighting the role of the cellular miRNA. Further, supplementation of the Dicer deficient cells with wild type MSC EVs was able to rescue the differentiation potential of the MSCs indicating the role of EV miRNA cargo in MSC EV functionality (Shirazi et al., 2021). Parallelly, our lab has shown that EVs derived from MSCs constitutively expressing BMP2 possess enhanced bone regenerative potential (Huang et al., 2020b; Huang et al., 2021). Results of these studies also indicated that in the EVs from MSCs constitutively expressing BMP2, a cluster of miRNAs that enhance the SMAD 1/5/8 signaling pathway was upregulated. This cluster included miR-424, 15a, 15b, 16, and 497. Of these miRNAs, miR-424, 15b and 497 have been shown in published studies to enhance bone repair by potentiating the BMP2 signaling cascade (Vimalraj et al., 2014; Xiao et al., 2015; Baptista et al., 2018; Pande et al., 2018; Ma et al., 2020).

Considering the importance of the miRNA cargo to MSC EV function and the influence of cellular state on EV miRNA composition, in this study, we sought to move the field of EV engineering forward by evaluating if pathway specific EV engineering can be accomplished by targeted expression of select miRNA in MSC EVs. Based on our results from the MSC EVs from BMP2 overexpressing cells and their miRNA cargo, we chose to generate functionally engineered EVs (FEEs) by generating MSCs that constitutively express miR-424 in an EV-specific manner. We hypothesized that, using EV-specific targeting sequences (Villarroya-Beltri et al., 2013), the identified miRNAs can be preferentially expressed in MSC EVs to generate FEEs that elicit pathway-specific activity. This approach differs from other miRNA delivery approaches such as RNA carriers or liposomes in that, apart from the delivered miRNA, the MSC EV itself possesses innate properties to promote repair. MSC EVs are readily endocytosed by target cells *via* the HSPG receptors on the plasma membrane (Huang et al., 2020a). The cargo is also polymerase-protected and delivered directly to the cell (Zhang and Chopp, 2016). As a proof of principle approach, we chose miR-424 as a candidate miRNA to target the SMAD 1/5/8 signaling pathway of the BMP2 cascade to enhance bone repair in a rat calvarial defect model.

## Materials and methods

### Cell culture

Human bone marrow derived stromal cells (HMSCs) were obtained from Lonza and cultured in αMEM basal media (Gibco) supplemented with 20% fetal bovine serum (FBS, Gibco), 1% l-glutamate (Gibco) and 1% antibiotic, antimycotic solution (Gibco) (Huang et al., 2020b). Primary bone marrow derived monocytes were isolated from mouse marrow as per our previously published protocols (Kang et al., 2020a; Kang et al., 2020b; Kang et al., 2022). The monocytes were differentiated into macrophages using macrophage colony stimulating factor (M-CSF) and subsequently polarized into pro-inflammatory (M1) or pro-reparative (M2) macrophages using LPS/IFNγ and IL-4 respectively as per previously published methodology (Kang et al., 2020a; Kang et al., 2020b; Kang et al., 2022).

### Targeted expression of miR-424 in HMSC EVs

EV-specific overexpression of miRNA-424 was carried out as per our published protocol (Mathew et al., 2023). Lentiviral particles containing a mammalian dual promoter vector that encodes for miRNA-424 followed by the EV targeting sequence under the control of EF1α promoter and a GFP marker under the control of SV40 promoter was custom generated by System Biosciences LLC and used for HMSC transduction. The vector was designed to target the 5p strand of the miRNA to the EVs. Positively transfected cells were selected for stable expression using puromycin. In parallel, a control vector without the miRNA was also transduced in HMSCs and similarly selected (referred to as GFP EV). The increase in expression of miR-424 3p and 5p fragments was verified by qRT-PCR from both cell and EV isolates as per our published protocols for cellular and EV miRNA quantitation (Huang et al., 2020b).

### Isolation and characterization of EVs

Functionally engineered EVs (FEEs) were isolated from the miR-424 expressing HMSCs as per standardized protocols (Narayanan et al., 2016; Huang et al., 2019; Huang et al., 2020b). Before isolation, the HMSCs were washed in serum free basal medium and cultured for 24 h in serum free basal medium. The conditioned medium was harvested and centrifuged at 3000xg to remove cellular debris. The medium was then concentrated five-fold (20% of original volume) using a 100 kDa cutoff spin filter (Millipore) and EVs were isolated from this concentrated medium using the ExoQuick TC isolation reagent (System Biosciences LLC). EVs isolated from the cells were resuspended in PBS at a concentration of 1.8 × 10^10^ particles/ml. The isolated EVs were characterized for the presence of markers CD63 (1/250, Abcam), CD81 (1/250, Abcam) and TSG101 (1/250, Abcam) by immunoblotting. Nano particle tracking analysis (NTA) was performed to obtain the size distribution of the isolated EVs.

### Endocytosis of EVs by HMSCs and macrophages

Primary bone marrow macrophages and HMSCs were cultured as described above in 96 well tissue culture plates (10,000 cells/well). GFP EVs and miR-424 EV were fluorescently labeled as per published protocols (Huang et al., 2020a; Huang et al., 2021). Dose dependent endocytosis was performed using GFP EVs and miR-424 EV using similar amounts of EVs. For qualitative experiments, the cells were cultured in 12 well dishes containing cover glass (50,000 cells/well). The cells were treated with 1.8 × 10^9^ fluorescently labeled GFP or miR-424 EV for 2 h at 37°C. The cells were fixed in formalin, permeabilized counterstained for actin using phalloidin TRITC (Sigma) and imaged using a Zeiss LSM 710 Meta confocal microscope.

### Calcium deposition assay

EV functionality was assessed at the level of MSC differentiation and BMP2 pathway activation by the miR-424 EV in comparison with vector control GFP EVs. MSC differentiation was assessed first by alizarin red staining. Briefly 100,000 HMSCs were seeded in 6 well culture dishes and subjected to osteogenic differentiation for 2 weeks using a combination of ascorbic acid, β-glycerophosphate and dexamethasone as per previously published protocols (Kang et al., 2022) in the presence/absence of control (GFP EVs) or miR-424 EV (3.6 × 10^9^ EVs/well). The culture medium was changed once in 2 days and the EVs were added to culture each time the medium was changed. Post 2-week culture, the cells were fixed and subjected to alizarin red staining to analyze the presence of calcium deposits qualitatively.

### Luciferase assay for BMP2 response element

A BMP2 response element luciferase assay was performed to analyze the triggering of BMP2 pathway. Briefly, HMSCs (50,000/well) seeded in 12 well plates were transfected with an SBE12 luciferase reporter plasmid that contains a BMP2 response element as per previously published methodology (Huang et al., 2020b). The transfected cells (48 h post transfection) were then treated with control or miR-424 EV (1.8 × 10^9^ EVs/well) as well as 250 ng/ml rh BMP2 (positive control). 48 h post transfection, the total protein from the cells were harvested and luciferase activity was measured using a BioTek microtiter plate reader as per published methodology (Huang et al., 2020b). For these experiments n = 4 replicates.

### *In vitro* osteoinductive gene expression assay

The ability of the miR-424 EV to trigger MSC differentiation was evaluated by qRT-PCR for osteoinductive gene expression. For this experiment, HMSCs were seeded in 12 well culture dishes and treated with miR-424 EV (24 h post seeding) as described above (n = 4). 72 h post treatment, RNA was isolated from untreated and treated wells and subjected to qRT-PCR for osteoinductive genes (VEGFa, TGFβ1, osterix, Runx2, BMP2, BMP6 and BMP9). Data were represented as mean fold change in gene expression over control.

### *In vitro* SMAD1/5/8 phosphorylation assay

A similar experiment to the one described above was performed to evaluate SMAD 1/5/8 phosphorylation by miR-424 EV. Here, total protein was isolated from the cells and immunoblotting was performed for phosphorylated SMAD 1/5/8 24 and 48 h post treatment with the EVs or rhBMP2. Tubulin served as loading control. The blots were imaged using a Licor Fluorescence imager. For quantitative measurements, an in-cell western was performed in 96 well plates (10,000 cell/well and 3.6 × 10^8^ EVs/well, n = 6).

### *In vitro* macrophage polarization assays

The effect of control and miR-424 EV on macrophages was evaluated *in vitro.* Primary mouse bone marrow derived macrophages were polarized towards inflammatory M1-like phenotype or reparative M2-like phenotype as described earlier in the presence/absence of control (GFP-EVs) or miR-424 EV. The expression levels of pro-inflammatory markers (IL-1β, TNFα and MCP-1) and reparative phenotype markers (Ym-1 and Arg-1) in M1 and M2 polarized macrophages respectively were analyzed by qRT-PCR as per established and published methodology (Kang et al., 2020a; Kang et al., 2020b; Kang et al., 2022).

### Rat calvarial bone defect model

All animal experiments were performed in accordance with protocols approved by the UIC animal care committee (ACC, Assurance No: A3460.01). All groups and time points contained 6 defects per group. Briefly, the rats were anesthetized intraperitoneally using Ketamine (80–100 mg/kg)/Xylazine (10 mg/kg). Using aseptic technique, a vertical incision was made in the head at the midline to expose the calvarial bone. The connective tissue was removed and two 5 mm calvarial defects were created bilaterally in the calvarium without dura perforation using a trephine burr. Each defect was covered by a collagen plug (5 mm diameter, 1 mm thickness) containing 9 × 10^9^ FEEs/control EVs/no EVs (PBS). Days 1, 3 and 7 post-surgeries, the tissue around the defects were extracted for qualitative and quantitative IHC. The tissues were fixed in neutral buffered formalin, embedded in paraffin and 5 μm sections were stained for Arg1 and iNOS as markers for reparative and inflammatory cells. The number of positive cells per field of view was counted using ImageJ software and used to obtain quantitative data (n = 6). Four- and 8-week post-surgery, the rats were euthanized by carbon dioxide asphyxiation and cervical dislocation. The calvaria were dissected, fixed in neutral buffered 10% formalin and scanned using a μCT scanner. The data from the scan was quantitatively analyzed using a custom MatLab software to obtain BV/TV data for all the groups and time points. The samples were then decalcified in 10% EDTA solution, embedded in paraffin, and 10 μm sections were subjected to H&E staining and immunohistochemistry (IHC) for bone morphogenetic protein 2 (BMP2, 1/250, Abcam), bone sialoprotein (BSP, 1/250, Abcam), dentin matrix protein 1 (DMP1, 1/250, Santacruz Biotechnology) and osteocalcin (OCN, 1/250, Abcam).

### Statistics

The normal distribution of the data obtained from the experiments was evaluated using the Shapiro-Wilk test. For experiments involving two groups, student’s *t*-test with a confidence interval of 95% was utilized. For the experiments involving comparison of more than two groups, one-way ANOVA was performed with a confidence interval of 95%. Pairwise comparisons were performed using Tukey’s *ad hoc* test with a confidence interval of 95%.

## Results

### Characterization of engineered EVs

**FIGURE 1:**
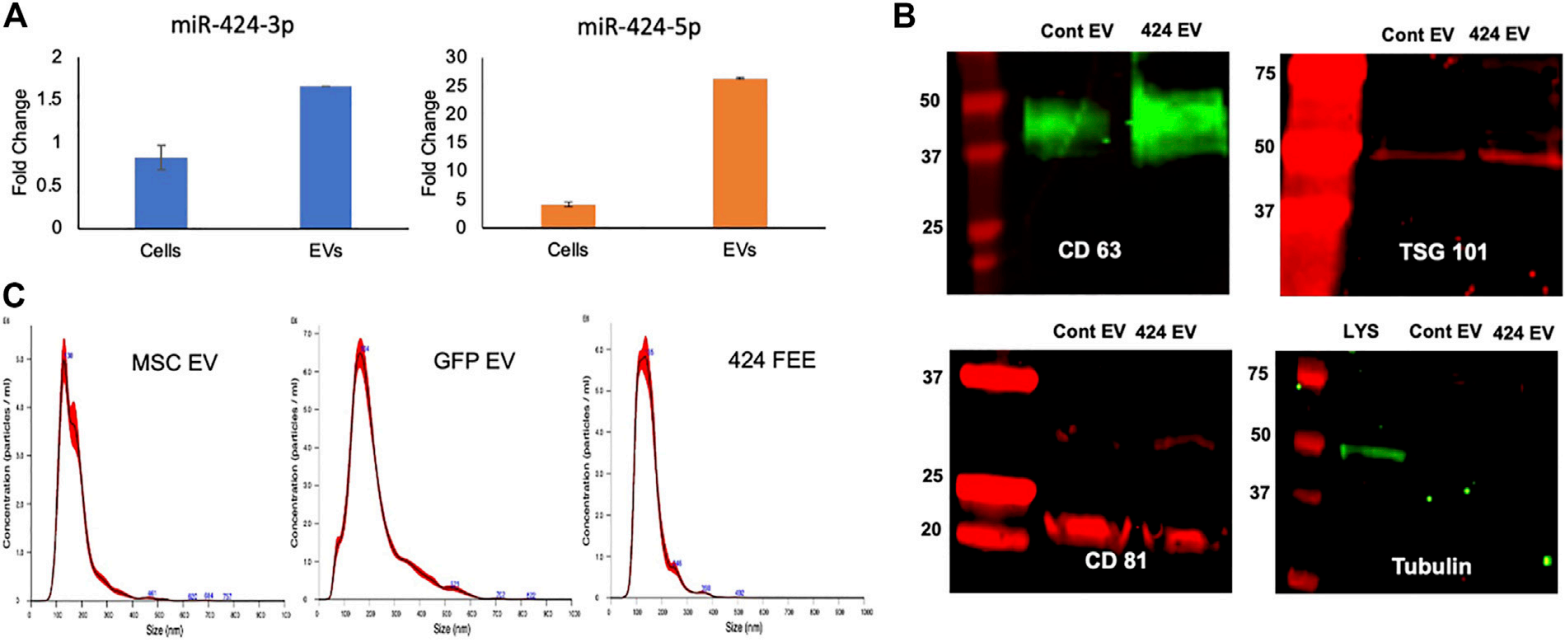
Characterization of the engineered EVs. **(A)** Fold change in the expression levels of the 3p and 5p strands of miR-424 in cellular extracts and EV extracts. Data show mean fold change ( ± SD) in expression with respect to vector controls. **(B)** Immunoblots of EV membrane proteins CD63 and CD81 and Intra EV protein TSG101 in control and engineered EVs. Tubulin was used as a control for absence of cellular protein in the EV lysates. Note the expression of tubulin in the cellular lysate (LYS) and not in the EV lysates. **(C)** NTA plots of EV size distribution indicating that no significant change occurred in EV size distribution in control and engineered EVs compared to naïve MSC EVs.

The expression levels of miR-424 3p and 5p strands were verified by qRT-PCR with respect to EVs from vector control (GFP EV). Figure 1A shows these results. A differential expression pattern of the strands between cellular and EV expression was noted indicating the EV-specific targeting of the miRNA 5p strand. The expression of both membranous (CD63, CD81) and intra EV (TSG101) marker proteins was verified by immunoblotting. The absence of intracellular proteins was verified by probing for tubulin using cell lysate as a control. Results presented in Figure 1B indicate that both vector control and miR-424 EV express EV marker proteins. The size distribution of the isolated EVs was evaluated by nanoparticle tracking analysis (NTA). Results indicated a similar size distribution between naïve MSC EVs, vector control GFP EVs and miR-424 EV indicating that EV-specific overexpression of miR-424 does not alter EV physical properties, marker expression and size distribution.

### Endocytosis of engineered EVs

**FIGURE 2:**
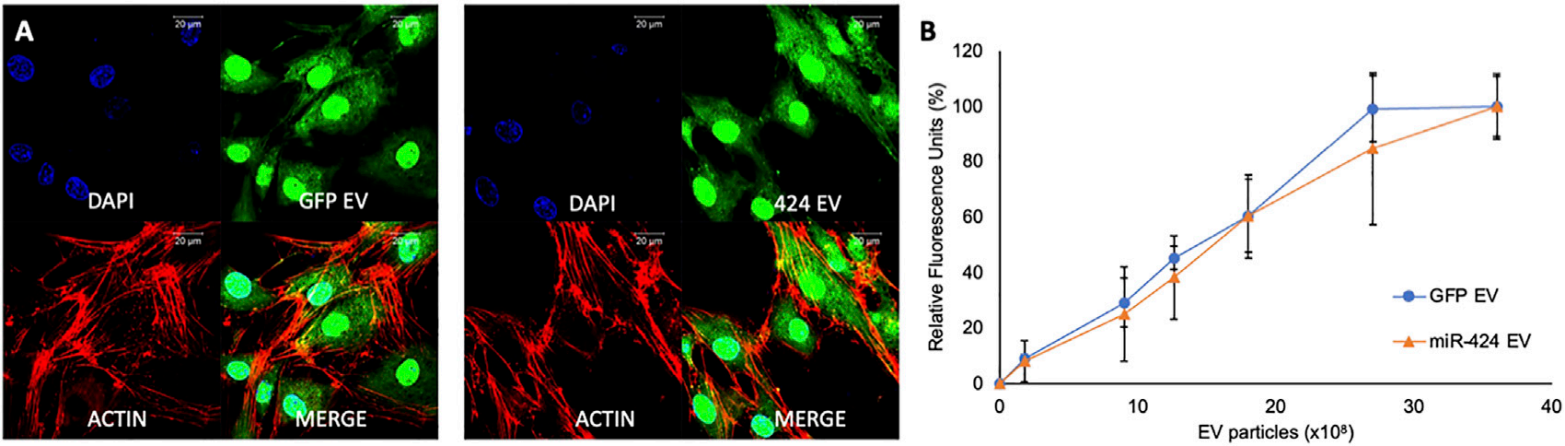
Endocytosis of EVs by MSCs. **(A)** Representative confocal micrographs of fluorescently labeled (green) control and 424 EVs endocytosed by HMSCs. Red represents actin counter stain, and the nuclei are stained blue with DAPI. Scale bar represents 20 μm. **(B)** Dose-dependent endocytosis of fluorescently labeled control and 424 EVs by HMSCs. No significant difference was observed between the ability of the two EVs to be endocytosed by HMSCs. Data are represented as mean ( ± SD).

We have published previously that MSC EVs are endocytosed by multiple cell types in a dose dependent and saturable manner (Huang et al., 2020a). Therefore, we verified if altering the EVs by either transfecting the vector or the miRNA construct alters its endocytic ability. The ability of the engineered EVs to be endocytosed by MSCs was verified qualitatively and quantitatively by immunocytochemistry and quantitative fluorescence measurements. Figure 2 shows the results of these experiments. Figure 2A shows the endocytosis of fluorescently labeled (green) control (GFP-EVs, left panel) and miR-424 EV (right panel) by HMSCs. The cells were counterstained using phalloidin-tritc for actin labeling and the nuclei were stained using DAPI. Figure 2B shows a dose dependent and saturable endocytosis of fluorescently labeled control and miR-424 EV by HMSCs.

### Osteoinductive properties of the engineered EVs (*In vitro*)

**FIGURE 3:**
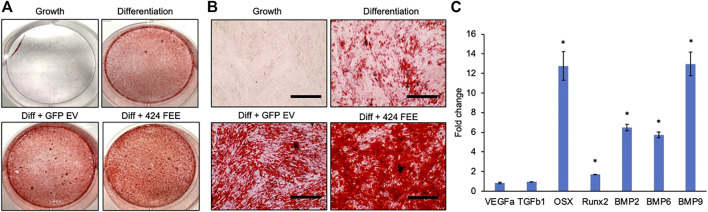
Osteoinductive property of the engineered EVs. **(A)** Macroscopic images of alizarin red stained HMSCs cultured for 14 days under the influence of osteogenic differentiation medium with/without control or engineered EV supplementation to analyze calcium deposition. Red staining denotes presence of calcium ions. Cells cultured under growth medium served as background control. **(B)** Microscopic (10x) images of the groups in **(A)**. Scale bar represents 100 μm. Note the increased presence of calcium in the EV treated groups and the highest level of calcium presence in the 424 EV group. **(C)** Fold change in expression levels of osteoinductive genes measured by qRT-PCR in 424 EV treated HMSCs (72 h post treatment) with respect to control group. Data are represented as mean fold change ( ± SD). * represents statistical significance with respect to control measured by students *t*-test, *p* < 0.05. Note the significant increase in the expression levels of osteoinductive growth and transcription factors.

miR-424 EV were generated to trigger osteogenic differentiation of naïve MSCs by enhancing the activity of the SMAD 1/5/8 signaling cascade. This property was evaluated *in vitro* qualitatively and quantitatively. First, the ability of the EVs to enhance the differentiation of HMSCs was verified by evaluating calcium deposition under the influence of osteogenic differentiation medium. Figure 3A shows the results of alizarin red staining of control and miR-424 EV treated HMSCs under differentiation conditions after 2 weeks in culture. Macroscopic (3A) and microscopic (3B) evaluation indicates that miR-424 EV promote enhanced calcium deposition under differentiation conditions compared to controls (differentiation conditions and differentiation conditions with control GFP EVs). Undifferentiated cells did not show the presence of calcium deposits. Following this, the ability of the EVs to induce the expression of osteoinductive genes was evaluated by qRT-PCR following 72 h of treatment of HMSCs with miR-424 EV compared to control. Results presented in Figure 3C shows that miR-424 EV triggered a significant increase (denoted by *) in the expression levels of several osteoinductive genes that include growth and transcription factors.

**FIGURE 4:**
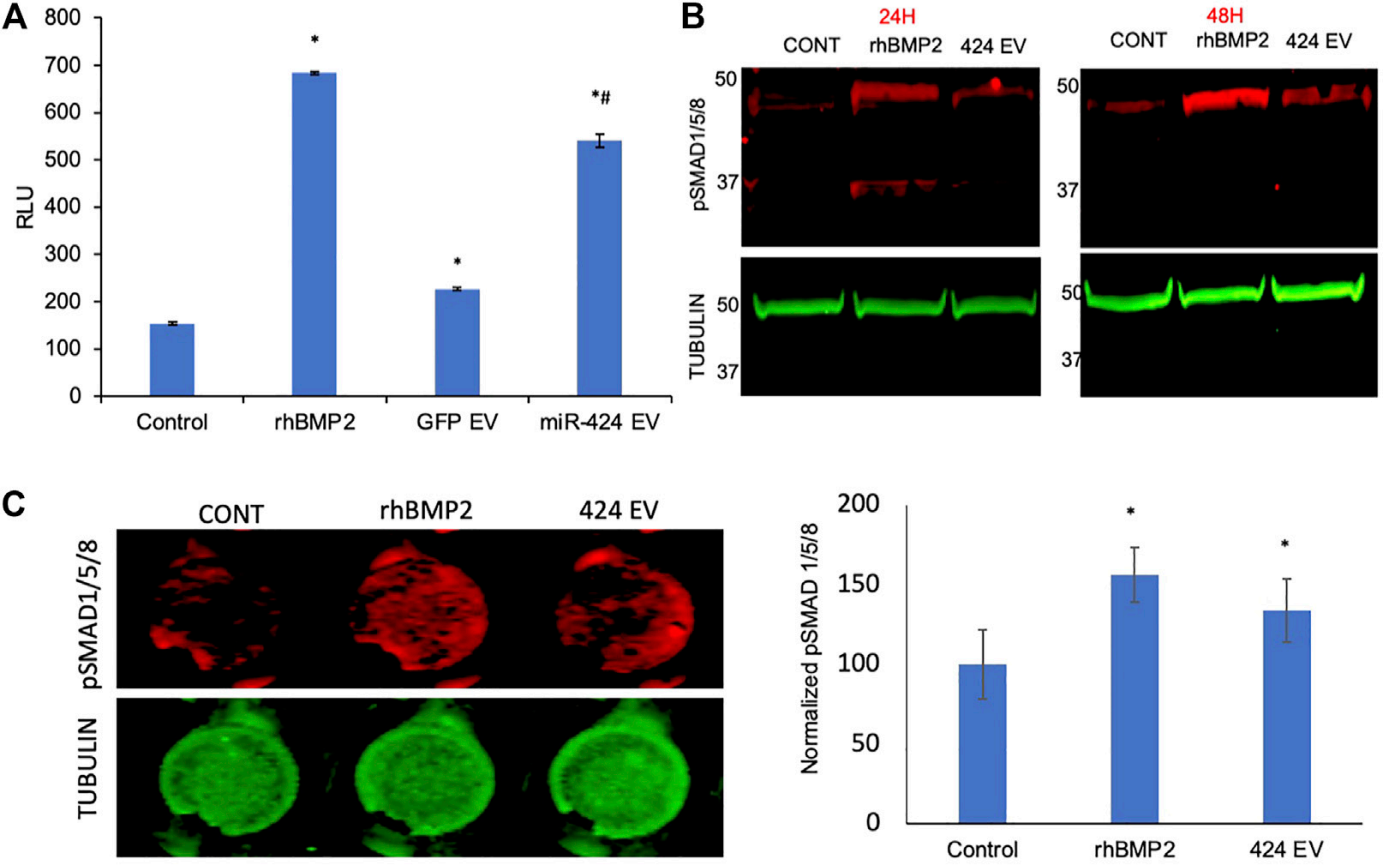
Engineered EVs trigger the BMP2 signaling cascade *in vitro*. **(A)** Graph representing luciferase activity in HMSCs transfected with a BMP2 reporter luciferase plasmid. Data are represented as mean luciferase activity (RLU, ± SD). Recombinant BMP2 protein (rhBMP2) was used as positive control. Note the significant increase in luciferase activity in 424 EV treated group compared to both untreated and vector controls. * represents statistical significance with respect to untreated control and # represents significance with respect to vector control (GFP EV) as measured by Tukey’s *post hoc* test following ANOVA. **(B)** Immunoblots showing the presence of phosphorylated SMAD 1/5/8 protein at 24 and 48 h after treatment with rhBMP2 (positive control) or 424 EVs. Tubulin was used a loading control. Note the increase in the presence of phosphorylated SMAD 1/5/8 in the 424 EV treated group compared to untreated controls. **(C)** quantitative in-cell western for the presence of phosphorylated SMAD 1/5/8. The image on the left is a representative image of pSMAD1/5/8 expression and the corresponding tubulin expression in a 96 well plate assay. The graph represents quantitation of the fluorescence from the wells using the LICOR Odyssey imager normalized to tubulin. * represent statistical significance with respect to the control group as measured by Tukey’s test post ANOVA.

To verify the induction of the SMAD 1/5/8 signaling cascade, we first evaluated the activity of the pathway using a luciferase assay that incorporates the SBE12 BMP2 response element. Our laboratory has utilized in earlier studies to evaluate the triggering of this pathway (Huang et al., 2020b). Figure 4A shows the results of this experiment. As is evident from the figure, positive control rhBMP2 triggered increased luciferase activity 48 h post treatment compared to untreated controls. The only other group to trigger significant increase in luciferase activity was the miR-424 EV group. This increase was statistically significant (n = 4, *p* < 0.05, Tukey’s test post ANOVA) when compared to untreated control group (*) as well as the control EV group (#). miR-424 belongs to a cluster of miRNAs that enhance endogenous SMAD 1/5/8 phosphorylation (Vimalraj et al., 2014; Xiao et al., 2015; Baptista et al., 2018; Pande et al., 2018; Ma et al., 2020). Therefore, we performed immunoblotting for detecting phosphorylated SMAD 1/5/8 in HMSCs treated with miR-424 EVs. rhBMP2 was used as a positive control for these experiments. Results presented in Figure 4B show that 24 and 48 h post treatment with either rhBMP2 or miR-424 EV, SMAD phosphorylation was increased. Quantitative measurement of this phenomenon was performed using an in-cell assay with tubulin as an internal control. Images and the corresponding normalized quantitation (n = 6) presented in Figure 4C indicate that miR-424 EV treatment significantly enhanced the presence of phosphorylated SMAD 1/5/8 in HMSCs. Taken together, the results presented in Figures 3, 4 collectively indicate that miR-424 EV can trigger osteogenic differentiation of HMSCs *in vitro* by potentiating the BMP2 signaling cascade.

### Immunomodulatory properties of the engineered EVs

**FIGURE 5:**
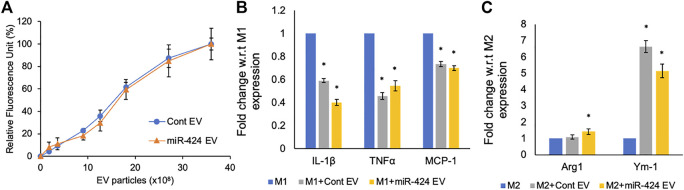
*In vitro* Immunomodulatory properties of the engineered EVs. **(A)** Graph representing the dose dependent endocytosis of fluorescently labeled control and 424 EVs by primary mouse bone marrow derived macrophages. No significant differences were observed. **(B)** Mean (+/-) fold change in expression levels of pro-inflammatory markers in M1 polarized macrophages in the absence/presence of control or engineered EVs. **(C)** Mean (+/-) fold change in expression levels of pro-reparative markers in M2 polarized macrophages in the absence/presence of control or engineered EVs. * represents statistical significance with respect to control measured by Tukey’s test post ANOVA.

MSC derived EVs possess inherent immunomodulatory properties that are designed to reduce inflammation by negatively regulating pro-inflammatory cytokines. Therefore, it is important for engineered EVs to maintain this property whilst also possessing enhanced pathway-specific activity they are engineered to target. We compared the activity of the miR-424 EV along with control EVs on primary mouse bone marrow derived macrophages. Both the control and miR-424 EV were dose dependently and saturably endocytosed by macrophages (Figure 5A). When macrophages were polarized to M1 phenotype in the presence/absence of EVs, both control and miR-424 EV triggered similar responses by significantly reducing the expression levels of IL-1β, TNFα and MCP-1 (Figure 5B). When similar treatment was evaluated on M2 polarized macrophages, both control and miR-424 EV significantly enhanced Ym-1 expression indicating similar *in vitro* activity on macrophages.

### *In vivo* evaluation of miR-424 EV

**FIGURE 6:**
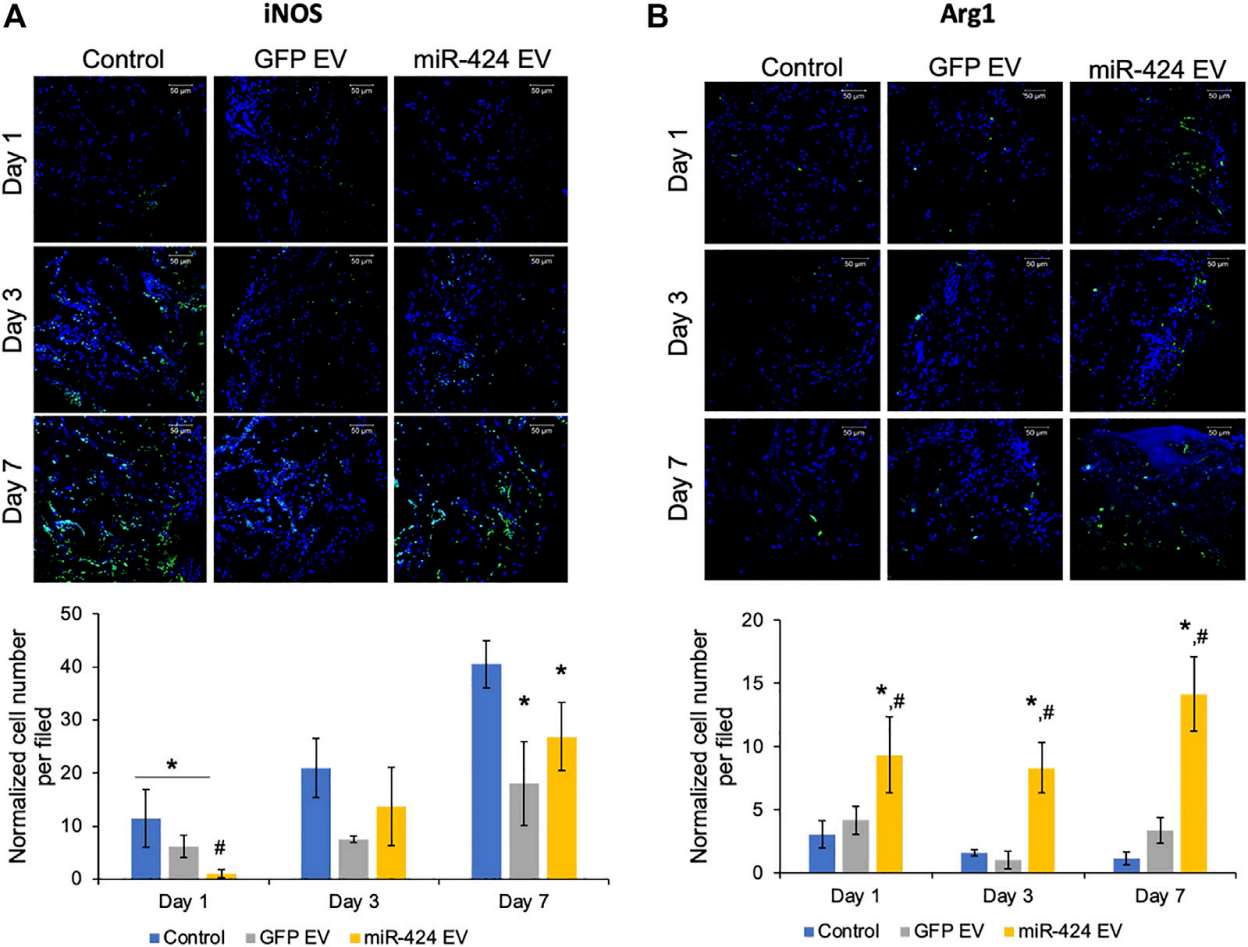
*In vivo* Immunomodulatory properties of the engineered EVs. **(A)** Representative confocal micrographs of calvarial wound beds at days 1, 3 and 7 post wounding immunostained for iNOS inflammatory macrophage marker (green) and counter stained with DAPI nuclear stain (blue) followed (below) by quantitation of average number of cells expressing iNOS per field of view. Note the significant reduction in iNOS expressing cells at days 1 and 7 in the EV treated groups and the significant reduction in 424 EV group compared to both control and control EV groups at day 1. **(B)** Representative confocal micrographs of calvarial wound beds at days 1, 3 and 7 post wounding immunostained for Arg1 reparative macrophage marker (green) and counter stained with DAPI nuclear stain (blue) followed (below) by quantitation of average number of cells expressing Arg1 per field of view. Note the significant increase in Arg1 expressing cells at days 1, 3 and 7 in the 424 EV treated group compared to both control and control EV groups. * in both graphs represents significance with respect to control and # represents significance with respect to control EV group measured by Tukey’s test post ANOVA.

We evaluated two different properties of miR-424 EV. First, we evaluated the immunomodulatory properties of the engineered EVs *in vivo* by performing immunohistochemical evaluation of pro-inflammatory marker iNOS and pro-reparative macrophage marker Arg1. The representative images for post-operative days 1, 3 and 7 for iNOS and Arg-1 and their respective quantitation are provided in Figures 6A,B respectively. Results presented in Figure 6A show that miR-424 EV significantly reduced the number of iNOS positive cells at day 1 compared to untreated control group as well as the GFP-EV group. However, while both EV groups reduced the number of iNOS positive cells at days 3 and 7 compared to controls, no significant differences were observed between them at these time points. On the other hand, miR-424 EV significantly enhanced the number of Arg1 positive cells at compared to both control groups at all time points.

**FIGURE 7:**
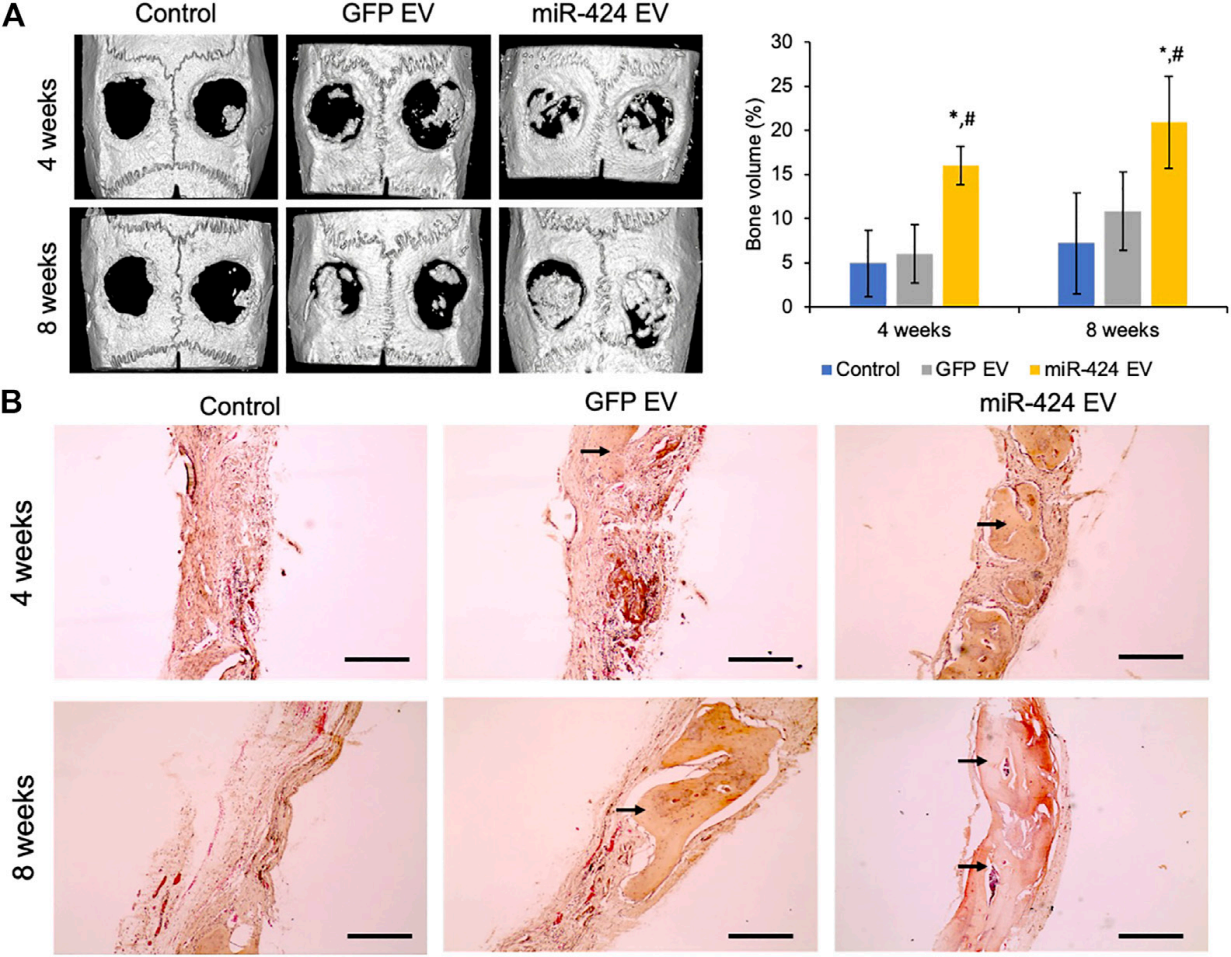
*In vivo* osteoinductive properties of the engineered EVs. **(A)** Representative μCT images of calvaria from control and EV treated groups after 4 and 8 weeks post wounding followed (right) by volumetric quantitation of bone formation in the defects. Note the significant increase in bone volume in the 424 EV treated groups compared to both controls. *represents significance with respect to control and # represents significance with respect to control EV group measured by Tukey’s test post ANOVA. **(B)** Representative micrographs (10x) of sections of decalcified calvaria stained with H&E stain. The arrows point to the presence of bone in the defects. Note the increased levels of bone in the 424 EV treated calvarial sections. Scale bar represents 100 μm in the micrographs.

**FIGURE 8:**
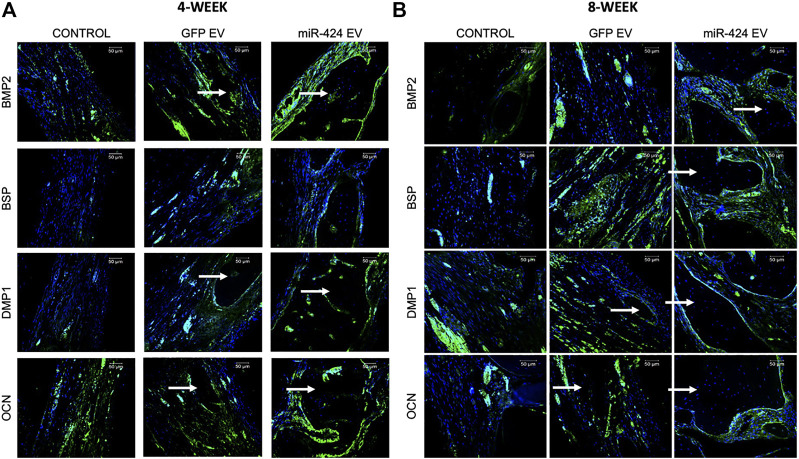
Immunohistochemistry of calvarial sections. **(A,B)** Representative confocal micrographs of calvarial sections from 4 to 8 weeks samples respectively immunostained for BMP2, BSP, DMP1 and OCN proteins. Scale bar represents 50 μm in all images.

Quantitative μCT was performed to evaluate bone regeneration 4- and 8-week post-surgery. Representative images presented in Figure 7A show increased bone formation in the miR-424 groups at 4 weeks compared to other control groups. Volumetric quantitation shown in Figure 7A shows that miR-424 EV triggered a significant increase in bone volume in the defects at both 4 and 8 weeks indicating their ability to enhance bone repair. Histological evaluation by means of H&E staining (Figure 7B) corroborated the μCT results with the miR-424 EV treated group showing increased presence of bone (arrows) compared to the control groups at 4 and 8 weeks. Finally, IHC was performed to evaluate the expression levels of osteogenic proteins in the defects. Results presented in Figure 8 indicate the expression of BMP2, BSP, DMP1 and OCN in representative sections from all groups at 4 and 8 weeks. While the increased presence of bone tissue in the miR-424 EV group is evident from these images, due to the varying degrees of the presence of connective tissue, the expression levels could not be quantitatively discerned. However, it is evident from the images that both EV treated groups show enhanced expression of the markers compared to the control group.

Collectively, the results presented in Figures 6–8 indicate that the engineered miR-424 EV possess immunomodulatory properties on par with control EVs, show enhanced propensity to trigger/recruit reparative macrophages (Arg1 positive cells) and promote enhanced bone formation.

## Discussion

This manuscript explores the possibility of EV engineering through targeted, EV-specific expression of select miRNA candidates for pathway-specific therapeutic activity. We have approached this study as a proof-of-principle approach using bone repair as a model system and the BMP2 signaling cascade as the target pathway. Bone is the second most transplanted organ in the human body second only to blood. While the use of growth factors such as BMP2 is approved for bone regenerative applications in several countries around the world, there are multiple complications arising from the use of BMP2 such as ectopic bone formation, severe inflammation, hematoma and wound dehiscence (reviewed in (James et al., 2016)). Therefore, we sought to engineer the properties of MSC EVs for potentiating the endogenous BMP2 cascade without the need for the use of growth factor.

For this study, we chose miR-424 as a candidate based upon our prior research that indicated that this miRNA featured among a cluster of miRNAs that are significantly up-regulated in the EVs of HMSCs when BMP2 was constitutively expressed in them (Huang et al., 2020b). Prior studies by other groups in the osteoblast system as well as in other organ systems have also identified that miRNA-424 positively regulates the BMP2 pathway by negatively regulating SMAD7 and SMURF1 (Grunhagen et al., 2015; Baptista et al., 2018; Li, 2020) both of which are negative regulators of the BMP2 signaling cascade. Furthermore, miRNA-424 has been identified as an anti-inflammatory miRNA in the nervous system with demonstrated efficacies in reducing microglial activation and neuronal cell apoptosis (Zhao et al., 2013). We have also recently demonstrated the activity of miRNA-424 engineered EVs in treatment of ischemic retinal diseases (Mathew et al., 2023).

The results presented in this study indicate that miRNAs can be specifically expressed in EVs without affecting the physical EV characteristics as well as their ability to be endocytosed by target cells. Our results show that there is no significant difference in the endocytosis profile of the engineered EVs compared to controls in both MSCs and macrophages. While we have not tested the endocytosis profiles of these EVs in other cells types involved in the bone wound bed such as endothelial cells and fibroblasts, we hypothesize based on the results presented here and the results reported previously by our group on MSC EV endocytic properties (Huang et al., 2020a), that they will remain unaltered. We believe that this is an important observation of this study from a translational perspective that indicates that EVs with altered miRNA cargo possess the same characteristics in terms of EV marker expression, size distribution and uptake by target cells.

From a functional perspective, the engineered EVs enhanced the osteogenic differentiation potential of naïve HMSCs compared to controls. In the presence of osteogenic differentiation medium, alizarin red expression in groups treated with the engineered EVs showed enhanced calcium deposition macroscopically and microscopically. Gene expression analysis focusing on the expression of osteoinductive genes indicated that the engineered EVs were able to trigger enhanced expression of these genes even in the absence of differentiating conditions indicating that these EVs were able to trigger the osteogenic differentiation pathways of naïve HMSCs. miRNA-424 is a potent activator of the SMAD1/5/8 pathway that governs BMP2 signaling (Grunhagen et al., 2015; Baptista et al., 2018; Li, 2020). In a luciferase assay that measures the activity of BMP2 signaling by using a BMP2 response element in HMSCs, the engineered EVs triggered significantly increased luciferase activity compared to control EVs and in line with positive rhBMP2 growth factor control indicating the ability of the engineered EVs alone to enhance pathway-specific activity in HMSCs. Immunoblotting showed and quantitative in-cell western assays revealed that when HMSCs were treated with the engineered EVs, enhanced levels of phosphorylated SMAD1/5/8 could be detected further confirming the pathway-specific activity of the engineered EVs. These *in vitro* experiments show that the engineered EVs possessed the pathway-specific activity they were designed to target. The SMAD 1/5/8 phosphorylation is an intermediate step in the signal transduction cascade related to BMP2 ligand/receptor signaling. By enhancing this step using engineered EVs, we have attempted to potentiate the endogenous BMP2 cascade without the requirement of increased ligand presence (BMP2). This method of pathway-specific intervention may be beneficial in relation to clinical translation by avoiding ectopic effects of growth factor mediated approaches.

When the engineered EVs were evaluated *in vivo* in a rat calvarial defect model, we were able to observe a significant increase in regenerated bone volume in the groups treated with the engineered EVs compared to controls confirming their osteoinductive potential. Histology confirmed this observation by showing enhanced bone presence in the defect areas. When the sections were examined by IHC for osteoinductive protein presence, an increased expression of these proteins were observed in connective tissues of the EV treated groups. However, due to the variability in the presence of connective tissue and the increased presence of bone in the histological sections of the engineered EV group, the expression levels of these proteins cannot be quantified accurately. This remains a drawback of this study and future studies will have to focus on extracting the total protein from these tissues pre decalcification and performing ELISA based quantitation to evaluate the presence of osteoinductive and mineralizing ECM proteins. While these assays confirm osteoinductive property of the engineered EVs, further studies are required to confirm the direct link between miRNA-424 in the EVs and the observed functionality. We also envision a possibility where the entire cluster of miRNAs that target the BMP2 cascade may be up regulated in the EVs when we manipulated the source MSCs. While qRT-PCR results have not shown this in our experiments, an informatics-based sequencing analyses of the control and engineered EV miRNA composition may be required to completely characterize the effect of this type of manipulation. In loss of function experiments, miRNA-424 is reported to be involved in dendritic cell differentiation and pro *versus* anti-inflammatory switch (Zyulina et al., 2021). However, loss of function studies in relation to bone formation and osteogenesis have not been performed to identify the significance of miRNA-424 to these processes. While our experiments show the benefits of increased presence of this miRNA, to fully understand its function knockdown experiments and/or experiments with antagomirs need to be performed to characterize the functional role of this miRNA. These are possible next steps that arise for the results presented here.

MSC EVs possess an inherent anti-inflammatory property and it is important to ensure that these characteristics of MSC EVs remained unaltered post EV engineering. One of the reasons behind the choice of miRNA-424 was also its reported anti-inflammatory activity (Rosa et al., 2007; Li et al., 2022). Therefore, we evaluated the anti-inflammatory and pro-reparative properties of the engineered EVs with respect to naïve MSC derived EVs. Our results indicated that *in vitro,* no significant differences could be observed between the two EVs, but *in vivo* studies indicated that immediately following injury (day 1 post wounding), the engineered EVs significantly reduced the number of iNOS expressing cells compared to other groups indicating an enhanced anti-inflammatory activity consistent with other published studies. Furthermore, results also showed increased levels of Arg1 (a pro-reparative M2 macrophage marker) positive cells in the engineered EV treated groups in days 1, 3 and 7 post-wounding indicating a pro-reparative environment triggered by the EVs. The possible disparity between the *in vitro* and *in vivo* results could be a result of the complexity of the *in vivo* environment that contributes towards macrophage polarization. We believe that when treated directly on isolated macrophages, the engineered EVs may not possess a significant activity over the control EVs. However, their overall functionality *in vivo* may be governed by their activity on other cell types involved in the wound bed resulting in the triggering of a more reparative environment compared to the control EVs. The role of the engineered EVs in this scenario requires further studies to elucidate which pathway-specific activity on what cell type results in such the observed change and these studies represent prospective next steps towards understanding the breadth of functionality of these engineered EVs.

Overall, the results presented here highlight the possibility of targeting the BMP2 cascade for bone repair by means of miRNA-based EV engineering. The osteoinductive function of these EVs enhance bone repair by promoting host MSC differentiation as well as by reducing injury related inflammation. While these studies presented here use wild type animals, future studies should focus on models of aging and systemic inflammation to evaluate the dual effects of these EVs on inflammation and stem cell differentiation. We envision that this is a good step in the direction towards precision EV therapeutics. EV therapeutics, especially the use of MSC EVs is an exponentially growing field of regenerative medicine. While there is tremendous promise, the safety and efficacy of these approaches require rigorous testing. At present, clinicaltrial.gov lists several clinical trials that are underway that utilize MSC EVs as therapeutic agents in the United States. The results from these trials will provide an overview of the challenges and hurdles that need to be overcome for mainstream medicine to incorporate EV therapeutics. In the meantime, recent studies have focused on the use of natural compounds to enhance MSC activity and function (Bouhtit et al., 2022). Our group has also evaluated such compounds for regenerative dentistry (Kulakowski et al., 2017). While the use of such compounds show promise, they too require rigorous testing before clinical translation. From an EV therapeutics translational perspective, the engineered EVs offer the convenience of mass production of EVs with consistent properties and future research should focus on exploiting this property by evaluating bio-reactor systems and EV stabilization technologies for commercialization and product development.

## Data Availability

The original contributions presented in the study are included in the article/supplementary material, further inquiries can be directed to the corresponding authors.
